# Isolated Chronic Inflammation in the Posterior Mitral Annulus

**Published:** 2016-04-13

**Authors:** Ali Hosseinsabet, Sohyl Mansorian, Maryam Sotoudeh Anvary

**Affiliations:** *Tehran Heart Center, Tehran University of Medical Sciences, Tehran, Iran.*

**Keywords:** *Mitral valve*, *Inflammation*, *Echocardiography*

A 56-year-old patient presented to the outpatient clinic with dyspnea on exertion (functional class II) of several months’ duration. Physical examinations were unremarkable except for a systolic murmur in the apex. Also, electrocardiography was unremarkable. Transthoracic and transesophageal echocardiographic examinations revealed moderate to severe mitral regurgitation and an echo-free space (4 × 2 cm) with an echogenic margin in the posterior mitral annulus (Figure1, Videos1-3). According to these findings, the patient was referred for surgery. In surgery, the posterior mitral annulus contained a cystic mass filled with a yellow fluid. After the aspiration of this fluid and the removal of this cystic mass, this space was repaired and mitral valve replacement with a mechanical bileaflet prosthesis was done. Fluid culture was negative for microorganisms. The Zill-Nelson staining for mycobacterium tuberculosis was negative. Pathological examination of the fluid and the mass margin showed degenerative cells and chronic inflammation, respectively. Serology test for hydatid cysts was negative. Chronic inflammation containing degenerative cells and presenting as a cystic mass in the posterior mitral annulus is rare. At patient presentation, we found no specific etiology for this inflammation. Accordingly, this isolated chronic inflammation can be the cause of mitral regurgitation as a rare etiology.

**Figure 1:**
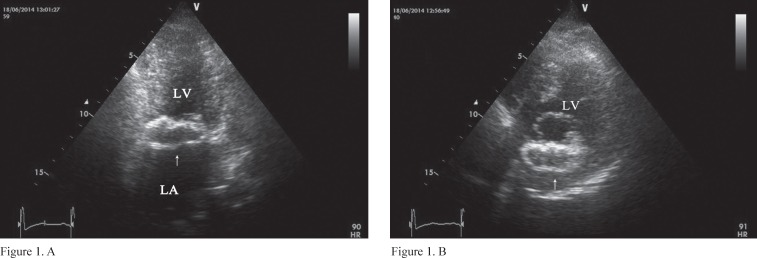
Echo-free space (arrow) in the posterior mitral annulus in the apical two-chamber view (A) and parasternal short-axis view (B).

LA, Left atrium; LV, Left ventricle

## Notes:

***This paper should be cited as: ***Hosseinsabet A, Mansorian S, Sotoudeh Anvary M. Isolated Chronic Inflammation in the Posterior Mitral Annulus. J The Univ Heart Ctr 2015;11(2):102-103.

## To watch the following videos, please refer to the relevant URLs:

Video 1. Echo-free space in the posterior mitral annulus in the apical two-chamber view

http://jthc.tums.ac.ir/index.php/jthc/article/view/588/464

Video 2. Echo-free space in the posterior mitral annulus in the parasternal short-axis view

http://jthc.tums.ac.ir/index.php/jthc/article/view/588/465

Video 3. Echo-free space in the posterior mitral annulus in transesophageal echocardiography (129°)

http://jthc.tums.ac.ir/index.php/jthc/article/view/588/466

